# Editorial

**Published:** 1860-02

**Authors:** 


### EDITORIAL.

We are very much obliged to our editorial brethren for the kind notices they have made of our first issue.

Many of them have represented the *Examiner* as the organ of the Medical Department of Lind University, with which the senior editor is connected. Though we do not complain of this, it is nevertheless untrue. The faculty of that Institution neither contribute a dollar to the support of this Journal, except as individual subscribers, nor control a single one of its pages. So far as the plan of instruction in that Institution accords with what we think the interests of the profession require, we shall commend it, but no farther. And we shall do the same thing by any other Medical College of which we may have occasion to speak, whether located in this city or elsewhere.

The *Chicago Medical Examiner* is the property of its editors, and as independent of all schools, clubs, or cliques, as any other medical periodical in the United States.

### MEDICAL INSTRUCTION IN CHICAGO.

The two Medical Colleges in this city are pursuing the even tenor of their way, each carrying out its own system of instruction successfully. The course in the Rush Medical College, which is still restricted to sixteen weeks, is rapidly drawing to a close; while that in the Medical Department of the Lind University continues until the first Monday in March. The number of Students in the latter Institution is 32. Its two departments, junior and senior, are carried on simultaneously with perfect regularity, and to the entire satisfaction of both divisions of the class.

We have recently received an announcement of the "Chicago Summer School of Medicine," by which we learn that Drs. G. K. Amerman, E. L. Holmes, J. P. Ross, E. Powell, H. W. Jones, W. C. Hunt, G. A. Mariner, and E. O. F Roler, have associated themselves together for the purpose of giving medical instruction to such students as may be induced to listen to them after the annual courses in the Colleges have closed. They promise one clinique, one lecture, and one recitation or examination each day, commencing on the first Monday in March, and continuing *sixteen* weeks. Terms $20, for the course, or $3.00 for single tickets.

All the members of this Association are young men of respectable attainments, and will do their best, both to instruct and please any who may place themselves under their charge.

We are authorized also to state that the Medical Faculty of Lind University will receive Students for the entire summer, and afford them every facility for pursuing the various branches of medical study, including hospital and dispensary cliniques, microscopy, and experimental physiology.

---

### EXPERIMENTAL PHYSIOLOGY.

We see it stated in a recent number of the *Med. and Surg. Reporter* of Philadelphia, that a claim had been set up to the effect that Professors A. Flint and J. C. Dalton, were the only teachers in the United States who illustrate their instruction in physiology by vivisections. The *Reporter* corrects this by adding the names of two other gentlemen in that city. And we further correct it by adding the names of Professors Deville and Hollister, of the Lind University, who have made some beautiful demonstrations by vivisections before the class during the present lecture term.

### Prof. DEVILLE'S LECTURE.

Some of our readers may think that we occupy too much space in the original department of the *Examiner* with a single article. We think, however, that in a Journal of 64 pages, it were better if we could have in every number *one* well written essay, in which some important subject is thoroughly investigated, than to fill the whole space with short and incomplete articles. It would contribute much more to the advancement of the science and literature of the profession, and yet leave room for an ample variety of matter. We are sure that the merits of Prof. Deville's lecture in the present number will fully repay a careful perusal by every reader.

---

### RUSH MEDICAL COLLEGE CLINIQUE.

### IMPORTANT NOTICE.

A regular clinique is given in the amphitheatre of the Rush Medical College every Saturday afternoon, by the Professor of Surgery, Dr. Daniel Brainard. Such cliniques are given in almost all the Medical Colleges throughout the country, and they are everywhere free for the attendance of regular practitioners, and especially for the *alumni* of the respective schools. Acting in accordance with this general custom, one of the Editors of this Journal, in company with another young practitioner of this City, (both being recent graduates of that College, and in good standing in the profession) went to the Rush Medical College, at the clinique hour for the 17th of December, and quietly seated themselves with the class in the lecture room. The Professor of Surgery soon entered with a patient, and began his clinique. He had proceeded but a few words when he discovered the two young practitioners alluded to, in the audience. He suddenly stopped, left the room, and in a few minutes sent them the following notice in his own handwriting, viz :

" Dr. Steele,

No person is allowed in this Clinique except members of the class and invited persons.

D. BRAINARD."

Of course the two gentlemen retired. But as this authoritative announcement from the President of that College, establishes a new order of things there; and especially a new relation between it and its numerous alumni, we have thought it proper to give the profession due notice of the fact; that any who might hereafter wish to spend an hour in the learned Professor's Clinique, should be careful to contrive some way to obtain a special "invitation" first. Of the propriety and *peculiar liberality* of this novel arrangement, we shall leave the profession to judge.

---

### CHICAGO COLLEGE OF PHARMACY.

We are glad to know that the efforts of a few of our leading and most enterprising Druggists, have resulted in establishing the above Institute upon a sure basis; and that during the progress of the first annual course of instruction they have shown themselves satisfied with the spirit that has called the enterprise into existence, by extending the hand of patronage. For we have never doubted that an effort to more fully develope the vast resources of our city, as well as elevate the standard of general professional requirements, would meet with a just and generous reward.

The members of the faculty are: James V. Z. Blaney, M. D., *Professor of Chemistry*; F. Scammon, M. D., *Professor of Pharmacy;* and John H. Rauch, M. D., *Professor of Materia Medica.*

---

### ILLINOIS STATE MEDICAL SOCIETY.

The next Annual Meeting of the State Society will be held at Paris, Edgar Co., the second Tuesday in May next. We call attention to this matter thus early, to remind the members of the profession of the duty they severally owe the Chairmen of the respective Committees. It is to be hoped that the following will be made a subject of special consideration:—

### TO THE MEDICAL PROFESSION.

At the Annual Meeting of the *Illinois State Medical Society*, held at Decatur on the first Tuesday in June last, the undersigned was appointed a Special Committee on the Medical uses of *Veratrum Viride.*

Being desirous of gaining all the information within reach, bearing upon the subject, so as to make as full and satisfactory a report as possible, I take the liberty of addressing you the following questions, and respectfully solicit an early answer.

1. Have you made use of *veratrum viride* in your practice? If you have:
2. In what form do you use it; (if the tincture; whose preparation?) and in what dose?
3. What are its effects?
4. In your opinion, what is its *modus operandi?*
5. What value do you attach to it as a remedial agent?
6. In what diseases have you found it most useful?

Give any other information upon the subject you may possess, and address A. Hard, M. D., Aurora, Kane County, Ill., as early as the first of March, 1860.

Yours, &c.,

*Aurora. Ill., Nov.* 28, 1859. A. HARD.

---

### FLORA OF ILLINOIS.

From a communication of Dr. George Vasey, of Ringwood, to the *Chicago Academy of Sciences*, we learn that about 200 species, including some 50 species of Cryptogamic plants, have been added to the Catalogue of the State; making some 1,200 species thus far observed.

---

### A FAVOR.

The unexpected demand for the first number of the *Examiner* has so far reduced the edition on hand, that we would esteem it a special favor if any ot those who have received the January number, and do not wish to become subscribers, would return the same to us, at our expense.

